# Proprioceptive Feedback and Brain Computer Interface (BCI) Based Neuroprostheses

**DOI:** 10.1371/journal.pone.0047048

**Published:** 2012-10-05

**Authors:** Ander Ramos-Murguialday, Markus Schürholz, Vittorio Caggiano, Moritz Wildgruber, Andrea Caria, Eva Maria Hammer, Sebastian Halder, Niels Birbaumer

**Affiliations:** 1 Institute of Medical Psychology and Behavioral Neurobiology and MEG Center, University of Tubingen, Tübingen, Germany; 2 TECNALIA, Health Technologies, San Sebastian, Spain; 3 Ospedale San Camillo, Istituto di Ricovero e Cura a Carattere Scientifico, Venezia Lido, Italy; 4 Department of Radiology, Klinikum Rechts der Isar, Technische Universität München, Munich, Germany; 5 McGovern Institute for Brain Research, Massachusetts Institute of Technology, Cambridge, Massachusetts, United States of America; Harvard University, United States of America

## Abstract

Brain computer interface (BCI) technology has been proposed for motor neurorehabilitation, motor replacement and assistive technologies. It is an open question whether proprioceptive feedback affects the regulation of brain oscillations and therefore BCI control. We developed a BCI coupled on-line with a robotic hand exoskeleton for flexing and extending the fingers. 24 healthy participants performed five different tasks of closing and opening the hand: (1) motor imagery of the hand movement without any overt movement and without feedback, (2) motor imagery with movement as online feedback (participants see and feel their hand, with the exoskeleton moving according to their brain signals, (3) passive (the orthosis passively opens and closes the hand without imagery) and (4) active (overt) movement of the hand and rest. Performance was defined as the difference in power of the sensorimotor rhythm during motor task and rest and calculated offline for different tasks. Participants were divided in three groups depending on the feedback receiving during task 2 (the other tasks were the same for all participants). Group 1 (n = 9) received contingent positive feedback (participants' sensorimotor rhythm (SMR) desynchronization was directly linked to hand orthosis movements), group 2 (n = 8) contingent “negative” feedback (participants' sensorimotor rhythm synchronization was directly linked to hand orthosis movements) and group 3 (n = 7) sham feedback (no link between brain oscillations and orthosis movements). We observed that proprioceptive feedback (feeling and seeing hand movements) improved BCI performance significantly. Furthermore, in the contingent positive group only a significant motor learning effect was observed enhancing SMR desynchronization during motor imagery without feedback in time. Furthermore, we observed a significantly stronger SMR desynchronization in the contingent positive group compared to the other groups during active and passive movements. To summarize, we demonstrated that the use of contingent positive proprioceptive feedback BCI enhanced SMR desynchronization during motor tasks.

## Introduction

Stroke survivors with chronic hand plegia and low scores in the Fugl-Meyer scale show limited residual muscle activity in the upper arm extensor muscles and no finger extension. Currently, there is no accepted and efficient rehabilitation strategy available in patients with chronic stroke and no residual hand movements. BCI systems could be a solution for those who suffered a stroke and need to rehabilitate a completely paralyzed limb and a damaged brain at the same time [1]. With this idea in mind, some groups explored motor imagery based therapy for motor recovery [2]. However, chronic stroke patients with motor impairment are usually treated with physiotherapy. Recently, robots as a way of facilitating treatment implying increased repetition and movement control were used [3], [4]. The control signal to activate the rehabilitation robots depends on the remaining muscle control. Force and kinematics sensors are used in robotics based motor rehabilitation as control signals whenever stroke survivors show residual movements to improve proprioceptive feedback [5], [6]. Alternatively, electromyography (EMG) can be used as a control signal alone or combined with force or kinematics sensors to improve movement detection [7]–[9]. For patients without any residual movement in the affected joints, EMG (if present) and electroencephalographic (EEG) signals, combined or alone, based BCI might be a non-invasive strategy that could be used to trigger robot movements and therefore close the loop between brain and effect (hand movements).

On the other hand, it has been widely demonstrated that visual feedback plays a key role in BCI training as in any other skill learning [10]–[14] and is the most used type of feedback. Recently, vibrotactile feedback [15], [16], auditory feedback [17] and robot assisted feedback control [18] have also been implemented. However, the specific type of feedback does not appear to be the critical factor for BCI performance [14]. Nevertheless, depending on the clinical application of the BCI and the remaining afferent pathways [10], [19], feedback can play a crucial role. Recently, BCIs approaches to motor rehabilitation in patients who suffered a stroke, proprioceptive afferent feedback becomes a key factor to close the loop and to produce some rehabilitation effects [1], [20], [21]. In these approaches proprioceptive feedback of passive movement of the paretic limb, was delivered to the patients after accomplishing a BCI task (normally driving a cursor on a screen to a target by modulating the sensorimotor rhythm of EEG and moving the limb with a prosthetic device). This passive approach can be called discrete proprioceptive BCI since a task has to be completed before receiving the proprioceptive feedback (several seconds delay), therefore the proprioceptive feedback was discrete (i.e. off-line) instead of continuous (miliseconds delay). On top of this, we know that passive motor trainings have not been shown to result in gains in motor function but active participation and volition seems necessary [8], [22], [23]. The control of a robot or prosthetic device and the feedback contingency are of vital importance to enable neuro-motor-rehabilitation. Here, we developed and tested in healthy participants an on-line proprioceptive BCI, closing the loop between brain, movement and proprioception. The difference between this system and previous studies [20], [21] consists of the online feedback being proprioceptive (feeling the hand moving) and visual (watching the hand moving) during voluntary brain control as opposed to online visual feedback only i.e. the feedback is represented by a cursor on a screen without concurrent movement, and passive movement with proprioceptive feedback and after successful cursor control only.

However, with at least some afferent pathways intact, the sensory information to the brain produced by moving the paretic limb engages remaining motor areas in the vicinity of the lesion to control the BCI. Since EEG has a limited spatial resolution it is difficult to separate activity from somatosensory cortex, premotor or motor cortex even using advanced spatial filtering methods [12], [24]. From previous work we know that passive movement affects frequency bands in a similar way but somewhat weaker than active movement and motor imagery [25], [26]. The afferent excitation of the sensorimotor brain through the robotic orthosis produces similar EEG frequency changes. Only preliminary data are available regarding the use of proprioceptive on-line BCI [27], [28]. Such BCIs could result in increasing and strengthening of the oscillations used for the BCI (i.e. improve in BCI control), or an opposite effect (decrease in BCI control comparable to distraction), or effects on other frequencies, electrodes or time points that do not affect the features of the BCI classifier. In order to test these alternatives, we developed a sensorimotor rhythm based on-line proprioceptive BCI, linking brain oscillations with a robotic hand orthosis and investigated the effects of proprioception on BCI control. 23 healthy participants separated in 3 different feedback contingency groups: contingent positive (n = 9), negative (n = 7) and sham (n = 7) feedback, were involved in the study. The participants performed five different tasks: (1) motor imagery without any feedback and no movement, (2) motor imagery with proprioceptive feedback of the BCI-dependent movement, (3) passive and (4) active movement without a BCI, and (5) rest, comparable with the major ingredients of rehabilitation therapies for movement disorders. Sensory motor rhythm (SMR) desynchronization/synchronization during each motor task with respect to inter-trial interval SMR was the performance measure and used as main dependent variable.

## Methods

### Experimental Procedure

23 healthy volunteers were recruited for the experiment. Participants were sitting in an upright position wearing a 128-channel EEG cap. The experimental protocol was approved by the ethics committee of the University of Tubingen, Medical Faculty. Participants provide their written informed consent to participate in this study. The hand of the participant was fixed to a hand orthosis which could be driven by the participants’ brain oscillations (the “closed” and “open” position of the hand was adapted to the individual range of motion). Participants were asked to perform 5 different tasks following 5 randomly presented auditory cues (the name of the task taped in advance (or from a taped recording of the voice of one of the experimenters):

1. *motor imagery without direct control* (MIT) of the orthosis i.e. the participant had to imagine to move the hand without moving the hand and with no movement of the orthosis (task1)
2. *motor imagery with direct control* (MIT&F) of the orthosis i.e. the hand motor imagery related brain oscillations drove the moving orthosis (task2)
3. *passive movements* of the orthosis i.e. the orthosis opened and closed the participants’ hand. The participant was asked not to perform any mental task. The orthosis movements are not linked to brain activity. (task3)
4. *active movement* i.e. the participant was required to actively open and close the hand attached to the orthosis and the orthosis followed the movement. (task4)
5. *rest* (task5)

The participants were separated in 3 different groups receiving 3 different feedback contingencies. Only during task2 participants used the EEG-based proprioceptive BCI to control the orthosis with opening and closing the hand motor imagery. The first group received contingent positive feedback (moving the orthosis with SMR desynchronization in task 2: 9 Participants), the second received contingent negative feedback (moving the orthosis with SMR synchronization in task 2: 8 Participants) and the third received sham feedback (the orthosis moved independently from brain activity but participants believed in their control: 7 Participants).

**Figure 1:**
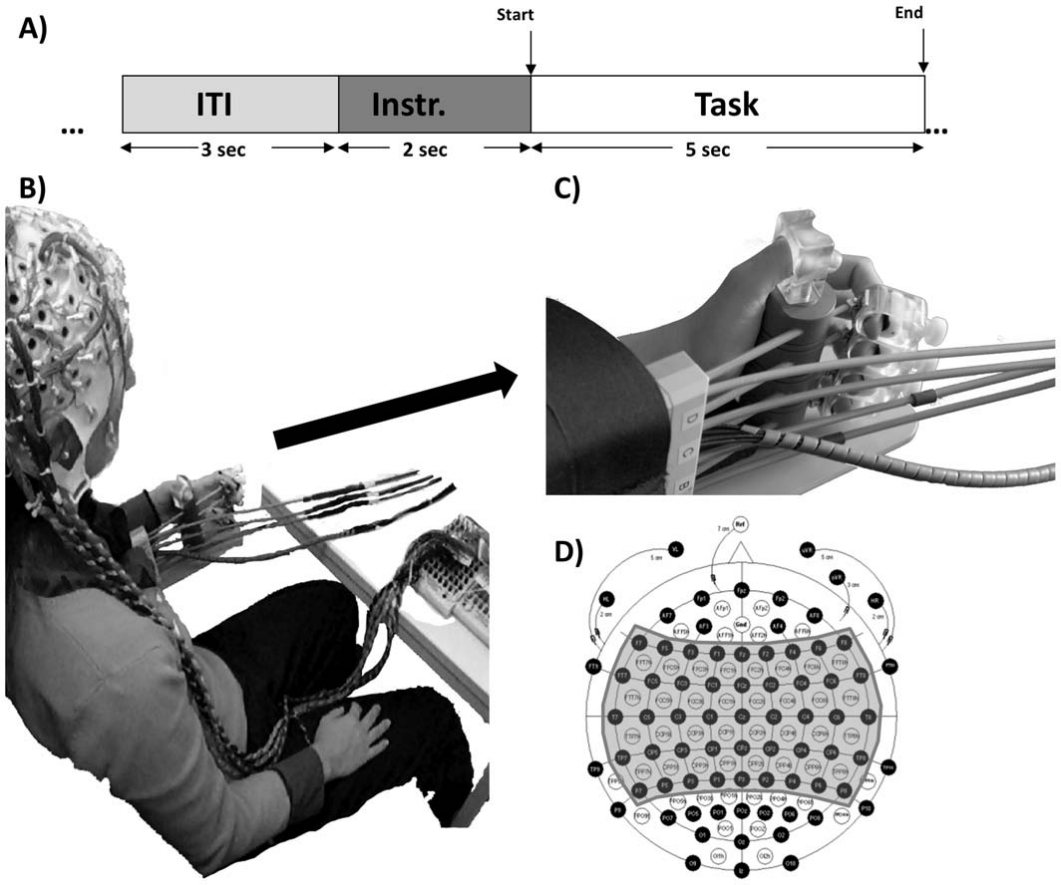
Experimental Design. A) Timing of an experimental trial. Each trial starts with a baseline of 3 seconds followed by an auditory instruction period. 2 seconds after the instruction a “Start” cue is presented and 5 seconds later an “End” cue. B) BCI. Participant wearing the 128 EEG channels cap seated with the hand attached to the orthosis showing the components used during all tasks C) Close look at the orthosis with the fingers attached. D) Schematic of the 128 channels and shaded in grey the 61 channels used during the experiments.

Two seconds after the corresponding auditory cue, a “GO” cue was presented and the participant performed the appropriate motor task for 5 seconds terminated by an auditory “end” cue (Fig. 1A). All auditory cues were normalized in pitch, length and volume. In task 1 and task 2 participants were asked to perform kinaesthetic motor imagery, i.e. imagine executing and perceiving the movements opening and closing the hand. During task 1 no feedback was presented to the participants in contrast to task 2, in which direct visual feedback of the hand, moving and proprioceptive feedback while the hand was moved by the brain-driven orthosis was provided (Fig. 1B). The participants performed 4 different training sessions at 4 different days completing 10 runs of 25 trials each. The participants had no prior BCI experience.

In Table 1 a demographic description of the participants is presented together with the oscillation type (sensorimotor rhythm synchronization S or desynchronization DS) used in each group to compute performance for the five different motor tasks. BCI performance except for task2 was computed off-line.

**Table 1:** Experimental protocol.

	Nr.	Age	Hand	Oscillation type used to compute BCI performance in all tasks/motor-modes
**CP**	9	26.6±4	9R	SMR Desynchronization
**CN**	8	26.5±5	8R	SMR Synchronization
**Sham**	7	26.2±2	7R/1L	SMR Desynchronization

Where “CP” indicates contingent positive, “CN” contingent negative and “Sham” sham feedback group. In the second column the number of participants of each group, in the third the average age and in the forth column the handedness. The last column indicates the oscillation type (sensorimotor rhythm synchronization or desynchronization) used to compute the BCI performance.

### Data Acquisition

EEG data were acquired using a BrainAmp 128-channel amplifier from Brain Products GmbH, Munich Germany. An EasyCap 128-channel EEG cap (modified 10–20 system) from EASYCAP GmbH, Herrsching, Germany was used for EEG data acquisition, referenced to the nasion, and grounded anteriorly to Fz. Only 61 EEG channels over the motor areas on both hemispheres were used recording from pre-motor, motor and parietal areas (Fig. 1C). Additionally, horizontal EOG on both eyes and vertical EOG on the right eye and EMG on both upper and lower arms for artefact correction was measured. Data were sampled at 500 Hz and transferred to a PC for storage and real-time signal processing using the BCI2000 platform (www.bci2000.org). EMG data were acquired using 8 bipolar Ag/AgCl electrodes (Myotronics-Noromed, Tukwila, WA, USA) placed on antagonistic muscle pairs; one close to the external epicondyle on the extensor digitorum (forearm extensor), the other on the flexor carpi radialis (forearm flexor), another on the external head of the biceps (upper arm flexor) and the last one placed on the external head of the triceps (upper arm extensor). The EEG and EMG electrodes impedance was always kept under 5 and 20 kOhm respectively.

### Orthosis

Each finger was moved individually using a DC−Motor M-28 (Kaehlig Antriebstechnik GmbH, Hannover, Germany) with worm gearhead for each finger. The motor drove a Bowden cable via cogwheel and cograil. A finger holder was mounted on the other side of each Bowden cable (Fig. 1C). Close to this finger holder an optical position sensor was mounted to detect the finger position independent of the bowden cable tolerance and elasticity. Strain gauges were placed on the Bowden cables near the fingers to detect the finger force in order to regulate the motor force to zero (no friction) for trials with active movement. A closed and an open finger position were predefined individually for each volunteer depending on their hand and finger size.

The BCI system determined the orthosis position and velocity and the device transmitted its actual position and velocity to the host computer upon request. Once the BCI system sends a position and a velocity command, the orthosis would then initiate a movement to the given position with the given velocity. Movement stopped when either the current position was identical to the position command sent by the BCI system (as set in the most recent position command), or when the velocity command was set to zero by BCI system. The direction of the movement was determined by the difference between current and desired position. As a physical connection between orthosis and host computer, a RS232 serial connection was used at a speed of 38400 bps. The BCI2000 two class classifier (motor imagery versus baseline) sent an output every 40 ms and five consecutive outputs for the same class were needed in order to send the orthosis a no-move (zero velocity value) or a move (positive velocity) command. This time filter was installed to avoid false positives and false negatives. During the sham feedback condition the BCI2000 output changed with a probability of 10%, i.e. when it was sending an output (e.g. moving) there was a probability of 10% that the next output would be the opposite (stop) and vice versa, requiring again 5 consecutive outputs of the same sign to change the movement status of the orthosis. This randomization of the output was identical to the averaged time participants from the contingent positive group achieved to move the orthosis during task 2.

### Signal Processing

The features to be used by the BCI platform were defined through a visual inspection of the R-squared values [29] obtained when comparing EEG activity during rest versus intention to move (hand open and close). The power in the electrodes and frequency bins with highest R-squared values were identified as customized sensorimotor rhythm (SMR) features, linearly combined with equal weights of −1 and used as input for a linear classifier. The result was normalized (zero mean, unit variance) with respect to the inter-trial interval period of each training run. We defined this final outcome as BCI output. Due to the weights used (i.e. −1), positive values of the BCI output during a trial reflected a SMR power spectrum decrease. In the online application, a center-surround local spatial filtering approach, in which a radial difference-of-Gaussians function was used to weight the electrodes at each spatial location, was applied to the EEG activity from each electrode. The spatial filtered EEG was modeled as an autoregressive (AR) process [30] of order 16 over a normalized sliding temporal window of 500 ms shifting every 40 ms and power spectral density of the AR-model for each electrode was computed to calculate the mean SMR-band power in each chosen frequency bin.

The BCI software maintained a history of the mean sensorimotor rhythm amplitude estimate from each trial and assigned this to a distribution representing observations for the two classes (rest or motor intention). The classification threshold, defined as the zero mean distance to the two distributions, was adaptive to account for changes in the shapes of these distributions over the course of training.

For EEG off-line analysis we performed a time-frequency analysis using a 1.142 s sliding window with an overlap of 26 ms. The event related spectrum perturbation was then calculated using Morlet transforms [31] with 3 cycles at lowest frequencies and 23.04 at highest, using the 200 ms time period from −1.5 to −1.3 s before the go cue as baseline for the event related spectra perturbation analysis. Power at 3 different frequency bins (8 – 12; 12 – 18; 18 – 25 Hz) was averaged during the 5 s after the “GO” cue for each motor task.

The EMG data were filtered using a high pass filter at 10 Hz, bipolarized, rectified and visually inspected. Trials presenting muscle activity during the resting task or absence of activity during active opening and closing of the hand were excluded from the event related spectrum perturbation analysis. On average, 8% of the EEG data acquired had to be rejected due to presence or lack of muscle activity during the experiment.

### Study Design

One EEG-screening was performed the day before the first training session and was used as a calibration session to identify the best features (electrodes and frequency bins) to be used by the BCI classifier. In this screening session the participants were randomly presented with visual and auditory cues corresponding to 3 different tasks indicating to either relax (task 1), actively open and close the right (task 2) or the left hand (task 3). After a 5 s period performing the tasks a rest cue was presented indicating to stop. The inter-trial-interval time was randomized between 5 and 7 s. The participants underwent 4 to 5 runs of 25 trials. The features to be used by the BCI platform were defined through a visual inspection of the R-square [29] values obtained when comparing EEG activity during rest versus intention to move (hand open and close). The power in the electrodes and frequency bins with highest R-square values were identified as customized sensorimotor rhythm (SMR) features and used as input for the classifier.

The group matching was performed based on age, handedness and the R-squared values obtained comparing the distribution of data during the screening session rest versus hand motor imagery.

After the screening, a cursor control training was performed at the end of the same session, to familiarize the participants with the BCI. For the cursor control training session participants controlled the velocity in the Y axis of a cursor moving from left to right on the screen at a constant speed trying to reach a target presented at the right side of the screen. The participants performed 4 runs containing 12 trials. The participants arrived at 4 consecutive days to perform one session every day. In every session the participants were presented with the 5 different tasks described before.

### Performance Measures

We analyzed how the BCI output changes during the different tasks and investigated the effect of the feedback contingency on BCI control. In addition to the online classification translated into orthosis movements (task 2), we simulated the performance the participants would have obtained if the orthosis would have moved during every motor task in an online setup. For example, how would the brain activity elicited during passive movement have been classified, if the classifier set up for motor imagery (same electrodes and frequency bins) would have been used to move the orthosis. Furthermore, several performance measures indicating different aspects of the SMR modulation were calculated off-line for all the tasks:

- Percent of time the orthosis was or would have been moved during a trial. This performance measure reflects the ability of the participant to decrease or maintain the decrease of SMR power during a trial.
- Maximum consecutive time the orthosis was moving per trial. This measure represents the longest period of time the participant was able to decrease or continuously maintain SMR desynchronization within a trial without inetrruption (synchronization in the contingent negative group).
- Number of orthosis moving onsets switching from not moving to moving per trial. This measure reflects how many times the participant loses and regains control of the orthosis within a trial.
- Latency to the first onset of orthosis movement per trial. This measure represents the reaction time of the participant in producing an orthosis movement (SMR desynchronization).
- Classical performance measure of reaching target, i.e. position of the cursor at the end of the trial, considering a successful trial if the cursor was in the upper half of the screen and unsuccesful otherwise.

These performance measures were calculated simulating an online scenario before and after EEG and EMG artefact removal to explore the influence of data contamination and the importance of implementing on-line artefact removal filters. We assumed that the proprioceptive feedback is felt by the user as number of times they can make the orthosis switch from not moving to moving (number of orthosis moving onsets), how fast they can start moving the orthosis (onset latency), percent of time the orthosis is moving during the trial and maximum consecutive time they moved the orthosis. We expected to observe learning effects in the contingent feedback groups (negative and positive) during the two tasks involving motor imagery (with and without feedback).

### Statistical Analysis

For each of the performance measures an ANOVA with two repeated measures (task and session) and three groups (between group factor) was performed to study main effects (session, task, group) and interactions (session × task, group × task, group × session, group × session × task).

Levene’s tests for homogeneity of error variances among groups were applied for all combinations of tasks and sessions. For all performance measures there were none or only few violations of the Levene-tests. Since the number of participants was less than 10 in each group and the number of performed tests was 20, slight violations were ignored and the error variances were assumed to be homogeneous.

Mauchly’s tests for the sphericity were done for the repeated measures factors and in case sphericity was violated significance tests were Greenhouse-Geisser corrected.

In this study we furthermore performed several planned contrasts to separately identify effects between the sessions, tasks and groups.

To identify learning over the four different BCI sessions we performed an ANOVA of the sessions within each group for each task separately. Mauchly’s tests for the sphericity were done for the repeated measures factors and in case sphericity was violated significance tests were Greenhouse-Geisser corrected.

We performed an ANOVA of the tasks for each of the groups and Bonferroni-corrected pairwise comparisons to identify main effects of the factor “task” and the source of it. The performance measures of the different sessions were combined for this step of the analysis.

To study the differences between groups the performance measures of the different sessions were combined and for each task separately we performed an ANOVA of the groups and Bonferroni-corrected multiple comparisons.

## Results

**Figure 2:**
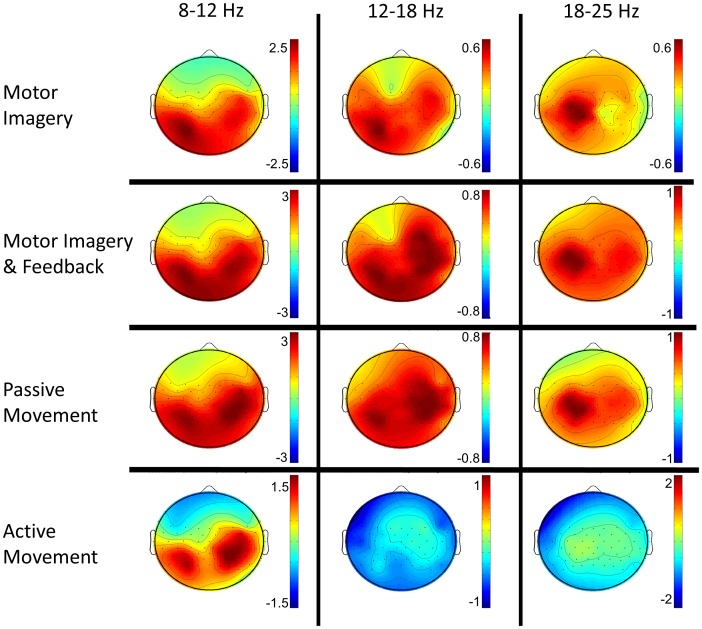
Motor task power distributions. EEG frequency domain power topoplots for each motor task averaged over all participants of the contingent positive group (all 9 participants were right handed and performed the task with the right hand). The EEG power from 3 representative frequency bins (8–12; 12–18; 18–25 Hz) was averaged over the 5 seconds of each task and subtracted from the one obtained using the same process during rest. Red and blue color correspond to event related desynchronization (ERD) and to event related synchronization (ERS) with respect to rest in dB. The activity distribution is very similar for all motor tasks presenting a clear contralateral motor and parietal activation and an ipsilateral motor-pre-motor activation.

EEG frequency analysis resulted in very similar event related synchronization and desynchronization (ERS/ERD) maps for all motor tasks when subtracting ERS/ERD power values during rest. A clear contralateral motor and parietal and an ipsilateral pre-motor activation common to most motor tasks and frequency bins was found. The active motor task presented more frequency power differences compared to the other tasks in the 8–12 Hz frequency bin (Fig. 2). This frequency range was the best frequency range to use in the BCI classifier after the screening session and resulted in the most consistent pattern of activation (Fig. 2).

### Overall Learning Effect

A statistical analysis was performed to study session effects (learning) for every motor task. A significant group effect was found for motor imagery alone (task 1) and motor imagery with feedback (task 2) for all performance measures (being always p<0.003) as expected except for the latency to the first orthosis movement onset (Table 2).

**Table 2:** Feedback “Type” effect on BCI control learning.

	Perc. Time	Max. Con.	Orth. Ons.	Lat. Ons.	Rea. Tar.
**MIT**	**F(1,14) = 21.89 p<0.001**	**F(1,14) = 19.29 p<0.001**	**F(1,14) = 43.57 p<0.001**	F(1,14) = 0.02 p = 0.881	**F(1,14) = 14.90 p<0.002**
**MIT&F**	**F(1,14) = 26.57 p<0.001**	**F(1,14) = 26.11 p<0.001**	**F(1,14) = 28.25 p<0.001**	F(1,14) = 1.56 p = 0.231	**F(1,14) = 22.27 p<0.001**

Feedback “type effect” when analyzing session effects (learning) for motor task motor imagery without feedback (MIT) (performance computed off-line) and with proprioceptive feedback (MIT&F) using 5 different performance measures for the contingent positive group (see text): percent of time the orthosis moved (Perc. Time); maximum consecutive time the orthosis moved (Max. Con); number of orthosis onsets (Orth. Ons.); latency to the first orthosis movement (Lat. Ons) and the classical reaching target (Rea. Tar). Statistically significant values (already Bonferroni-corrected) are marked bold. All were significant except Lat. Ons.

### Group Learning Effect

**Figure 3:**
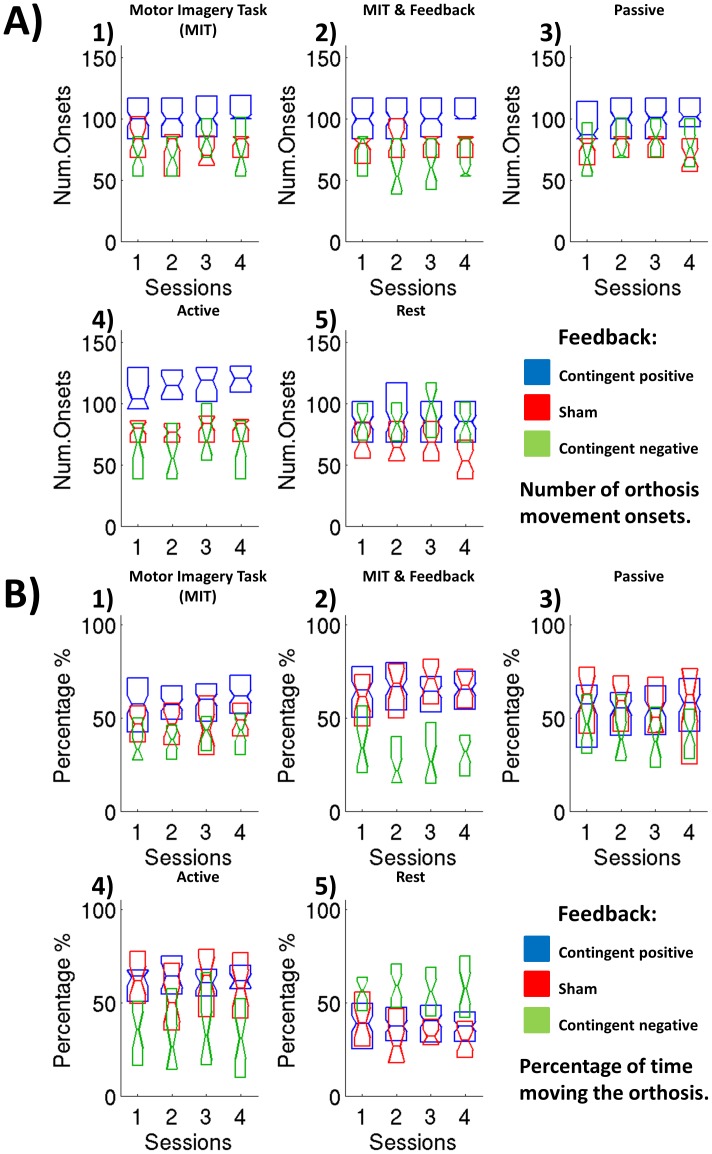
BCI performance using 2 different measures. The midpoint of each box corresponds to the median value and the upper and lower margines correspond to the 25 and 75 percentiles. Differences marked with an asterisk are statistically significant. A) Number of orthosis moving onsets per session for each group during motor imagery without any feedback, with proprioceptive feedback (orthosis moved through BCI) (MIT&F) (task 2), passive and active movements (with natural visual and proprioceptive feedback). The contingent positive group outperformed the other 2 groups significantly and shows a significant learning effect during motor imagery without feedback (MIT) (task 1). B) Percent time moving the orthosis per session for each feedback group in the different tasks. The contingent positive and sham feedback percent of time moving the orthosis is always significantly higher compared to the contingent negative group with the exception of the motor imagery task without feedback (MIT) (task 1). In this condition the contingent positive group showed significantly higher BCI performance compared to the other feedback groups.

When analyzing every feedback group separately (contingent Positive (CP), contingent negative (CN) and sham) for each movement task the only significant session effect (F(3,24) = 4.406, p = 0.0128) was an increase in number of onsets during motor imagery alone in the contingent positive feedback group only (Table 3 and Fig. 3). Despite of a positive trend in the learning curve during motor imagery with feedback (task2) (Fig. 3A), the high variance in performance led to no significant learning effect probably caused by the high performance level (ceiling effect).

**Table 3:** Statistical analysis of session effects (learning).

Nr. Orth.Ons.	MIT	MIT&F
**CP**	**F(3,24) = 4.406, p = 0.013**	F(3,24) = 2.041, p = 0.135
**Sham**	F(1.651,9.908) = 0.241, p = 0.771	F(3,18) = 2.081, p = 0.139
**CN**	F(1.910,11.458) = 1.092, p = 0.365	F(3,18) = 1.813, p = 0.181

Statistical analysis of session effects (learning) for motor task motor imagery without feedback (MIT) and with proprioceptive feedback (MIT&F) using the number of orthosis movement onsets per session as performance measure for each feedback group (contingent positive (CP), negative (CN) and sham). The statistics were performed on each group independently. Statistically significant values (p<0.05) (already Bonferroni-corrected) are marked bold.

### Individual Learning Effect

The learning effect was tested for every healthy volunteer independently comparing the first session performance to each of the other sessions using the Kruskal Wallis Test (non-parametric) Bonferroni corrected for multiple comparisons (data were mostly non-spherical). In the contingent positive feedback group 3 participants showed statistical significant increase in maximum consecutive and percent of time during motor imagery task alone and with proprioceptive feedback. The participants without significant increase (learning) showed high values of performance (ceiling effect). In the sham feedback group (non-contingent) and contingent negative feedback group no significant learning occurred in any of the participants.

### BCI Performance Group Differences for Tasks

When comparing group performance measures averaged over sessions for each motor task we observed that during motor imagery without feedback (no movement of the orthosis occurred) (task 1), the percent of time the orthosis would have been moved and the number of orthosis movement onsets were significantly higher (Bonferroni-corrected post-hoc-test) for the contingent positive compared to the contingent negative and sham feedback groups (Fig. 3.A, 3.B). The maximum amount of time the orthosis was/would be moving continuously per trial and the reaching target accuracy performance measures were significantly higher for the contingent positive compared to the contingent negative feedback group, and higher (but not significant) compared to the sham feedback group (Table 4). No significant difference was found when comparing sham and contingent negative feedback groups in any of the performance measures. Although in the contingent negative group participants moved the orthosis synchronizing their SMR brain oscillations and in the sham feedback group performance calculated offline (movement of the orthosis) was linked to desynchronization of SMR oscillations, both resulted in similar BCI performance.

**Table 4:** Statistical analysis on group differences.

	GroupsCompared	PercT	MaxC	NOns	Lat	ReachT
**MIT&F**	**CP-CN**	**0.0001**	**0.0001**	**0.0001**	0.599	**0.0001**
	**CP-Sham**	1.000	1.000	**0.004**	1.000	1.000
	**CN-Sham**	**0.001**	**0.0001**	0.093	0.396	**0.000**
**Active**	**CP-CN**	**0.002**	**0.006**	**0.0001**	0.144	**0.012**
	**CP-Sham**	1.000	1.000	**0.0001**	**0.004**	1.000
	**CN-Sham**	**0.019**	**0.025**	0.129	0.454	**0.034**
**MIT**	**CP-CN**	**0.0001**	**0.001**	**0.0001**	1.000	**0.004**
	**CP-Sham**	**0.027**	0.073	**0.0001**	1.000	0.210
	**CN-Sham**	0.317	0.313	1.000	1.000	0.317
**Passive**	CP-CN	0.341	0.416	**0.003**	1.000	0.786
	CP-Sham	1.000	1.000	**0.002**	0.627	1.000
	CN-Sham	0.216	0.147	1.000	1.000	0.309
Rest	CP-CN	**0.001**	**0.001**	1.000	**0.001**	**0.001**
	CP-Sham	1.000	1.000	**0.012**	0.027	1.000
	CN-Sham	**0.001**	**0.001**	**0.004**	**0.001**	**0.001**

Statistical analyses on the groups (contingent positive (CP), negative (CN) and sham) differences in performance averaged over sessions during motor imagery without feedback (MIT) and with proprioceptive feedback (MIT&F), active and passive movement and rest. The performance measures were the percent of time moving the orthosis (PercT), maximum consecutive time moving the orthosis per trial (MaxC), number of orthosis movement onsets (NOns), latency to the first orthosis Onset (Lat) and reaching target performance (ReachT) per session. Statistically significant values (p<0.05) (already Bonferroni-corrected) are marked bold.

During motor imagery with proprioceptive feedback (task 2) and during active movement alone (task 4), the percent of time moving the orthosis, the maximum consecutive time moving the orthosis and reaching target accuracy performance measures were significantly lower for the contingent negative group compared to the contingent positive and sham groups (Table 4), although we found no significant differences between the sham and contingent positive groups. On the other hand, as for the imagery task alone (task 1), during imagery task with proprioceptive feedback (task 2) and active movement (task 4) the number of orthosis movement onsets was significantly higher for the contingent positive compared to the contingent negative and sham feedback groups for the online simulation.

There was no significant difference during passive movement (task 3) between groups for any performance measure but the number of orthosis movement onsets, which in the contingent positive group showed higher values compared to the other 2 feedback groups (Fig. 1.A).

### Overall Task BCI Performance Differences within Every Group

We analyzed the difference in the off-line calculated performance for all tasks within each individual feedback group averaging all the sessions. When analyzing the contingent negative group we could not find any significant difference between motor tasks using any performance measure other than the difference between rest and active movement BCI performance difference. The same effect was found in the sham feedback group as well as a significantly higher maximum consecutive and percent of time moving the orthosis during motor imagery with proprioceptive feedback (task 2) when compared with motor imagery task without any feedback.

On the other hand, in the contingent positive feedback group, all the performance measures during motor imagery without and with proprioceptive feedback and active movement were significantly different (higher percent and maximum consecutive time moving the orthosis, number of orthosis movement onsets and reaching target, and lower for orthosis movement onset latency) compared to rest as expected.

During passive movement, the number of orthosis movement onsets was significantly higher compared to rest indicating that passive movement generated afferent brain activity affects sensorimotor brain oscillations resulting in a significant increase of orthosis movement onsets when using the proprioceptive BCI. Furthermore, a significantly higher number of orthosis movement onsets were found for active movement when compared to passive movement.

## Discussion

A significant group effect during imagery with and without feedback (task 1 and task 2) was found for all performance measures except for the latency to the first orthosis movement onset. Contingent positive group showed significantly larger difference between SMR power during rest (increase in power) and motor imagery tasks (decrease in power) and therefore higher BCI performance compared to the other 2 feedback groups. This finding suggests that after the go cue the time needed to move the orthosis was not significantly different between feedback groups and indicates that an initiation based therapy (after the first BCI mediated robot movement onset, this moves continuously along a predefined trajectory and independently of participants brain activity) would not show any significant difference in performance between feedback groups.

We observed a significant learning effect during motor imagery task with no feedback (task 1) indicating implicit learning (they did not receive any feedback during this task) present in the contingent positive feedback group only. The participants were trained and rewarded for motor imagery and therefore learning was expected to occur during task 2 only (motor imagery task with proprioceptive feedback). We also expected some learning during task 1 motor imagery with no feedback because the same task was repeated although feedback was only present in task 2.

As we can see in Fig. 3A and 3B the overall performance i.e. SMR desynchronization was higher for task 2 and task 4 on average, indicating a positive influence of proprioceptive feedback on brain activity and on BCI performance. However, there was no significant difference in the number of orthosis movement onsets between motor imagery with and without feedback in the contingent positive group. This result together with the longer time moving the orthosis during motor imagery with feedback implies similar processing resources to move the orthosis during motor imagery with and without feedback tasks but a higher capability of maintaining the SMR desynchronization when receiving proprioceptive feedback. Furthermore, feedback contingency seems to affect the resting network since the number of spontaneous onsets in the contingent positive group was significantly higher than the onsets of the sham group and similar to the onsets obtained by the contingent negative group during rest.

BCI learning effects (learning of ERD and ERS) were expected to be stronger for percent time and maximum consecutive time moving the orthosis in task 2 because the visual and proprioceptive feedback (orthosis moving) provides maximal information about correct or incorrect control of the BCI. The data indicate that participants of contingent positive group only learned to start moving the orthosis, i.e. to change from ERS to ERD.

For all the tasks except rest (motor imagery with and without feedback and active and passive movement), the performance was significantly higher for the contingent positive group in terms of orthosis movement onsets when compared to the other 2 groups. Furthermore, percent of time and maximum consecutive time were significantly higher in the contingent positive group too but only during motor imagery with feedback (task 2) (proprioceptive BCI).

In the contingent positive group only significantly higher performance was observed during passive movement when compared to resting BCI performance. These findings suggest that feedback contingency (proprioceptive stimulation paired with EEG SMR desynchronization) influences the motor network enhancing significantly SMR down- regulation. The use of the proprioceptive BCI assists to desynchronize the SMR rhythm during any motor related activity, i.e. the findings indicate that contingent proprioceptive BCI training only operates by priming and engaging a group of ecologically relevant brain regions related to imagery of a task, supporting the proposal of using an online proprioceptive BCI to induce neural changes. These changes could be used as a boosting effect for any passive or active physiotherapy of the same movement. However, although enhancing the SMR modulation during the passive mode, the BCI performance during the use of the online proprioceptive BCI, motor imagery and active movement was significantly higher than during passive movement alone, indicating that the effects would be significantly higher during active engagement in the motor task.

All three groups showed significant difference in performance between rest and active movement. However, the significant difference in performance between rest and the other motor tasks (except for active movement) occurs only in the contingent positive feedback group, which underlines the importance of contingent proprioceptive BCI.

The results extend experiments of (motor) skill learning to BCI-control, confirming the mechanistic similarity of the two and confirming animal experiments with BCI control of single neurons [32]–[34] and the hypotheses [35] demonstrating a tight time contingency of proprioceptive and visual feedback and the active-voluntary mode of instrumental learning as a prerequisite of learning. None of the two essential ingredients of skill learning is sufficient to improve motor learning: learning without immediate rewarding feedback is not possible and active-voluntary repetitive behaviour alone cannot secure learning if it lacks feedback.

### Conclusions

We investigated an online proprioceptive BCI system linking hand movements and brain oscillations, eliciting implicit learning effects and producing an increase in SMR related neural networks excitation during motor imagery, passive and active movement. We propose the use of the here described proprioceptive BCI as a potential motor rehabilitation tool to be used in paralyzed patients with residual proprioception (e.g. stroke patients).
